# Branch length estimation and divergence dating: estimates of error in Bayesian and maximum likelihood frameworks

**DOI:** 10.1186/1471-2148-10-5

**Published:** 2010-01-11

**Authors:** Rachel S Schwartz, Rachel L Mueller

**Affiliations:** 1 Department of Biology; Colorado State University; Fort Collins, CO, 80523-1878, USA

## Abstract

**Background:**

Estimates of divergence dates between species improve our understanding of processes ranging from nucleotide substitution to speciation. Such estimates are frequently based on molecular genetic differences between species; therefore, they rely on accurate estimates of the number of such differences (i.e. substitutions per site, measured as branch length on phylogenies). We used simulations to determine the effects of dataset size, branch length heterogeneity, branch depth, and analytical framework on branch length estimation across a range of branch lengths. We then reanalyzed an empirical dataset for plethodontid salamanders to determine how inaccurate branch length estimation can affect estimates of divergence dates.

**Results:**

The accuracy of branch length estimation varied with branch length, dataset size (both number of taxa and sites), branch length heterogeneity, branch depth, dataset complexity, and analytical framework. For simple phylogenies analyzed in a Bayesian framework, branches were increasingly underestimated as branch length increased; in a maximum likelihood framework, longer branch lengths were somewhat overestimated. Longer datasets improved estimates in both frameworks; however, when the number of taxa was increased, estimation accuracy for deeper branches was less than for tip branches. Increasing the complexity of the dataset produced more misestimated branches in a Bayesian framework; however, in an ML framework, more branches were estimated more accurately. Using ML branch length estimates to re-estimate plethodontid salamander divergence dates generally resulted in an increase in the estimated age of older nodes and a decrease in the estimated age of younger nodes.

**Conclusions:**

Branch lengths are misestimated in both statistical frameworks for simulations of simple datasets. However, for complex datasets, length estimates are quite accurate in ML (even for short datasets), whereas few branches are estimated accurately in a Bayesian framework. Our reanalysis of empirical data demonstrates the magnitude of effects of Bayesian branch length misestimation on divergence date estimates. Because the length of branches for empirical datasets can be estimated most reliably in an ML framework when branches are <1 substitution/site and datasets are ≥1 kb, we suggest that divergence date estimates using datasets, branch lengths, and/or analytical techniques that fall outside of these parameters should be interpreted with caution.

## Background

One of the major goals of phylogenetic systematics is to accurately estimate divergence dates among species and clades [1]. In addition to determining the timing of species' emergences [e.g. [2]], studies of divergence dates across a range of taxa have revolutionized our understanding of processes ranging from nucleotide substitution and selection [e.g. [3,4]] to patterns and processes of speciation [e.g. [5-7]]. Divergence dating has allowed investigators to determine environmental conditions leading to increased biological complexity [e.g. [2]], correlate rapid radiations with colonization of new habitats [e.g. [8]], identify the effects of environmental changes on species with different life histories [e.g. [5]], and determine the rate of evolution and timing of emergence of viruses such as Ebola and HIV [e.g. [9-12]]. Consequently, correct estimates of divergence dates are crucial for research across many fields of study.

Divergence date estimates were initially based on fossils; however, fossils are necessarily younger than the date of divergence [1]. Research in molecular divergence dating was initiated with the proposal of a molecular clock [13]. Subsequent research suggested that such a clock model is violated for distantly related species; however, local clocks for individual genes can be applied due to similarities within groups in their metabolic rate, generation time, DNA repair efficiency, and functional constraints on particular genes [4,14,15]. There have been many advances in divergence dating since the initial proposal of a molecular clock. Analyses allowing for rate variation over time (i.e. relaxed clock methods) range from non-parametric rate smoothing [e.g. [16]] and semi-parametric penalized likelihood [e.g. [17]] to highly parametric maximum likelihood [e.g. [15,18,19]] and Bayesian [e.g. [12,20,21]] methods. In many cases, such methods have resulted in significant changes to estimated divergence dates, and in some cases, they have helped resolve discrepancies between fossil and molecular dates [e.g. [22]]. All of these methods of analysis rely on correct estimation of branch lengths (i.e. average number of substitutions per site).

Underestimation of branch lengths can result from uncounted multiple substitutions at some sites [23,24]. Although multiple substitutions were previously thought to occur only for ancient divergences, variation in evolutionary rates across sites due to different functional constraints can produce multiple hits even for recent divergences [25-27]. When calibration points are set at deeper nodes, which is often the case due to limited availability of fossil or other calibration data, the underestimation of the number of substitutions on the calibrated long branch results in an underestimated substitution rate. When this rate is used to estimate divergence dates for shallower nodes, for which associated branch lengths were not underestimated, the underestimated rate results in nodes that are estimated as older than their true values [28].

Substitution models can be used to estimate unobserved substitutions; therefore, accurate specification of the substitution model plays a critical role in correct branch length estimation [28-30]. Substitution model misspecification can result from: (1) estimating insufficient parameters to fit the evolutionary process, and/or (2) estimating such parameters incorrectly. The former problem has been addressed in several ways, including incorporating rate heterogeneity across sites [26] and among lineages into models used in phylogenetic analyses [31-34]. More complex models can produce different branch length estimates compared to simpler models for the same dataset [35]. However, statistical power for estimating parameters is limited by amount of available data, and the addition of parameters further reduces such power [36]. The addition of imperfectly estimated parameters may have negative impacts on branch length estimation [30].

Although adequate models are necessary for accurate branch length estimation, they provide no guarantee of accurate estimates. Substitution models specify only relative rates among different types of substitutions, but the absolute, average rate of substitutions/site along each branch must still be estimated from the data. Thus, specific features of the dataset (e.g. sequence length, number of taxa), the individual branch (e.g. length, depth of position in the tree), and the overall tree (e.g. variation in branch lengths) all likely impact branch length estimation. However, the effects of these variables, alone and in combination, on branch length estimation have not been fully explored.

We used extensive data simulations to (1) systematically vary dataset size, branch length, and tree complexity, and (2) compare estimated branch lengths to known branch lengths to identify the determinants of estimation accuracy in both Bayesian and maximum likelihood frameworks. We examined model parameter estimates for all analyses to look for confounding effects of model misspecification. Finally, to determine how inaccurate branch length estimation may affect estimates of divergence dates, we reanalyzed an empirical dataset for plethodontid salamanders [37,38], based on our simulation results suggesting the conditions under which branch length estimates were most accurate, to determine how inaccurate branch length estimation may affect estimates of divergence dates.

## Results

### Baseline Bayesian branch length estimates

**Figure 1:**
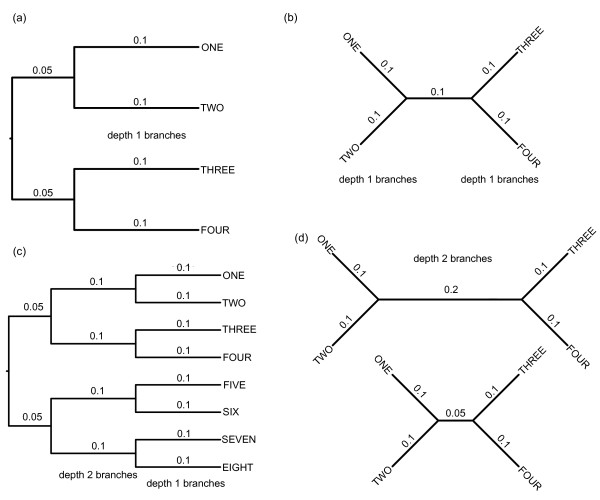
**Example trees used in simulations**. (a) Balanced 4-taxon trees with equal branch lengths used for basic data simulations. This tree shows one of the 11 sets of branches of different lengths. (b) Unrooted version of the tree in (a) used for branch length estimation. When the tree is unrooted it is clear that all branches are of equal length. (c) Balanced 8-taxon trees of equal branch lengths used to determine whether branch length estimation is affected by (1) the depth of the branch in the tree, and (2) the number of taxa. (d) Balanced 4-taxon trees with equal depth 1 branch lengths and the depth 2 branch half or double the length of the depth 1 branches. These trees were used for simulations to determine whether interactions among branch lengths affect branch length estimation accuracy.

**Figure 2:**
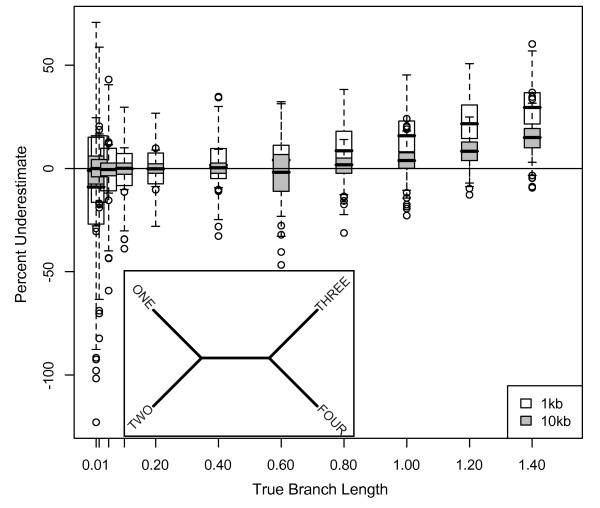
**Underestimate of Bayesian branch lengths for 4-taxon trees**. Percentage that branch lengths were underestimated for 1 and 10 kb datasets simulated on 4-taxon trees with equal branch lengths (inset) using the HKY model with a transition/transversion ratio of 2 and equal base frequencies, and analyzed using MrBayes with an HKY model, estimated model parameters, and the default exponential prior (mean = 0.1) on branch lengths. The box plot shows the range of misestimation across all branches and simulations; results were identical for single branches and total tree length.

The accuracy of branch length estimation varied with branch length. Using Bayesian methods to analyze 4-taxon trees with equal branch lengths (Figure 1), the length of longer branches was significantly underestimated, while the length of short branches was significantly overestimated (Figure 2). For the longest branches (1.4 substitutions/site), the median branch length underestimate was ~30% for 1 kb datasets (Figure 2 white boxes). For the shortest branches (0.01 substitutions/site), the median branch length overestimate was ~9%. Branch lengths were estimated approximately correctly in the range of 0.02-0.4 substitutions/site. For branch lengths greater than 0.6 substitutions/site, the rate of branch length underestimation increased linearly. Subsampling the data had no effect on these results. To show the full range of misestimation, the percent underestimate for all branches is shown in Figure 2; however, because error in one branch may lead to error in a connected branch, we also examined misestimation of (1) individual branches, and (2) total tree length. The median and range of misestimation for these measures were nearly identical to the median and range for all 400 branches for each set of simulations shown in Figure 2; thus, misestimation for all 400 branches is shown for this and other figures.

### Effects of dataset size on Bayesian branch length estimates

As dataset size increased, branch length misestimation decreased. A 10-fold increase in data reduced the median underestimation of branch lengths of 1.4 substitutions/site from 30% to 15%. Similarly, this increase in dataset size reduced overestimation of branch lengths of 0.01 substitution/site to 1%. (Figure 2 gray boxes). The pattern of increasing branch length misestimation with increasing branch lengths was consistent regardless of dataset size.

### Effects of number of taxa and branch depth on Bayesian branch length estimates

**Figure 3:**
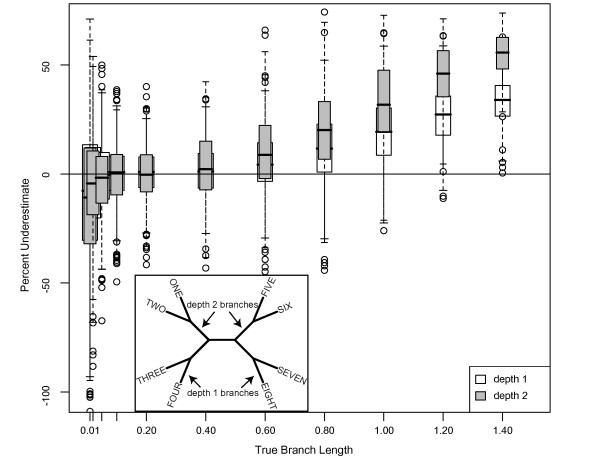
**Underestimate of Bayesian branch lengths for 8-taxon trees**. Percentage that branch lengths were underestimated for 1 kb datasets simulated on 8-taxon trees with equal branch lengths (inset) using the HKY model with a transition/transversion ratio of 2 and equal base frequencies, and analyzed using MrBayes with an HKY model, estimated model parameters, and the default exponential prior (mean = 1/10) on branch lengths. Branches with depth = 1 and depth = 2 (see inset) were evaluated separately (white and gray boxes, respectively).

Error in branch length estimation increased with branch depth. The lengths of depth 1 branches in the 8-taxon tree (Figure 3 inset and white boxes) were misestimated at rates comparable to depth 1 branches in the 4-taxon tree (Figure 2 white boxes). However, depth 2 branches in the 8-taxon tree were underestimated at significantly greater rates for longer branch lengths (Figure 3 inset and gray boxes). Branches of 1.4 substitutions/site at depth 2 were underestimated by approximately 55%, compared to 30% underestimation for depth 1. The pattern of a linear increase was consistent for depth 1 and depth 2 branches, with the depth 2 branches having a steeper slope (Figure 3). These results suggest that the position of the branch on the tree affects branch length estimation; however, the number of taxa in the dataset does not appear to affect branch length estimation for branches of the same depth on the tree. Subsampling the data had no effect on these results. Additionally, fixing the tree topology in this analysis did not affect either the median branch length estimate (less than 1% different from the unfixed topology in all cases) or the variance in the estimates.

### Effects of branch length heterogeneity on Bayesian branch length estimates

**Figure 4:**
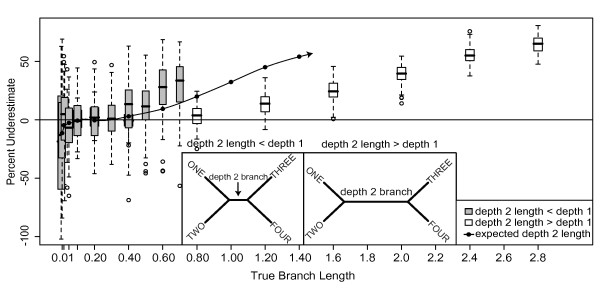
**Underestimate of Bayesian branch lengths on unequal branch length trees**. Effects of unequal branch lengths on branch length estimation. Gray boxes are the percent underestimation of depth 2 branch lengths for 4-taxon trees with the depth 2 branch length = half the depth 1 branch length (left inset). White boxes are the percent underestimation of depth 2 branch lengths for 4-taxon trees with the depth 2 branch length = double the depth 1 branch length (right inset). Depth 2 branch lengths were expected to be underestimated at the same rate as depth 2 branch lengths of 8-taxon, equal-branch-length datasets (mean underestimation shown as filled circles). Half-length depth 2 branches (gray boxes) were underestimated at a significantly higher rate than expected (filled circles). Double-length depth 2 branches (white boxes) were underestimated at a rate significantly lower than expected (filled circles and extrapolating from the trend of underestimation (spline interpolation line)). The range of depth 2 branch lengths examined in this analysis was dictated by the range of depth 1 branch lengths examined in the overall study (0.01-1.4 substitutions/site).

Estimation of a single branch length was affected by the length of the other branches in the tree. When one branch of the tree was half the length of the other branches, the length of this branch was more underestimated than expected (Figure 4). Similarly, when one branch was double the length of the other branches, the length of this branch was less underestimated than expected (Figure 4). These results suggest that the rate of underestimation for the "majority" branch lengths exerts a "pull" of the rate of underestimation of the "unique" branch length, such that the unique branch length is underestimated at a rate more similar to the rate of underestimation for the majority branches in the tree. However, due to our study design, we cannot discriminate whether this effect is due to the majority branches being the majority, being depth one branches, or some other cause.

The expected rate of underestimation for depth 2 branches for these 4-taxon simulations was derived from underestimates for depth 2 branches for 8-taxon simulations (Figure 4, filled circles). Because shorter branches were estimated more accurately than longer branches (Figure 2), when the depth 2 branch of the 4-taxon simulation tree was half the length of the four depth 1 branches, we expected the depth 2 branch to be less underestimated than the depth 1 branches in the tree. However, this half-length depth 2 branch was actually underestimated at a rate similar to the depth 1 branches of the 4-taxon tree, which was significantly higher than the rate of underestimation for depth 2 branches for the 8-taxon equal branch length tree (Figure 4 gray boxes v. filled circles). When the depth 2 branch was double the length of the four depth 1 branches in the 4-taxon simulation tree, the depth 2 branch was more underestimated than the depth 1 branches in the tree, as predicted; however, the depth 2 branch was underestimated at a rate significantly lower than depth 2 branches of the same length for the 8-taxon equal-branch-length tree (Figure 4 white boxes v. filled circles).

### Effects of branch length prior on Bayesian branch length estimates

**Figure 5:**
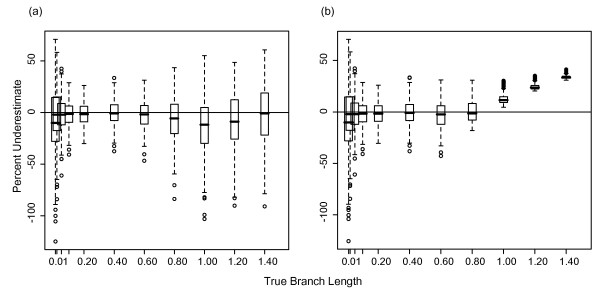
**Underestimate of Bayesian branch lengths with different branch length priors**. (a) Percentage that branch lengths were underestimated for 1 kb datasets simulated on 4-taxon trees with equal branch lengths using the HKY model with a transition/transversion ratio of 2 and equal base frequencies, and analyzed using MrBayes with an exponential prior on branch lengths of mean = 1. (b) Identical to (a) but analyzed with a uniform prior on branch lengths (bounds of 0-1).

The prior probability distribution also affected branch length estimation. When we estimated branch lengths using an exponential prior with a mean of 1 for the 4-taxon tree, most branch lengths were overestimated, with the degree of overestimation ranging from 1 to 12% (Figure 5a). For branches ≤ 0.2 substitutions/site for the 4-taxon HKY datasets, branch length estimation was similar in accuracy when using a uniform prior as when using the default exponential prior with mean equal to 0.1. However, whereas branches ≥ 0.4 substitutions/site were increasingly underestimated when using the default prior, branch lengths ≤ 0.8 substitutions/site were estimated approximately correctly when using the uniform prior (Figure 5b). However, for branch lengths > 0.8 substitutions/site, the underestimation increased linearly and more rapidly than with the default prior. Consequently, branches of 1.4 substitutions/site were underestimated by approximately 35% (Figure 5b), which is slightly greater than the underestimation for the same branch lengths when using the default prior (30%; Figure 2). Results for 8-taxon trees were similar; depth 1 results using a uniform prior were comparable to the 4-taxon results, and depth 2 branches ≥ 0.8 substitutions/site were underestimated at a higher rate than depth 1, as expected from the 8-taxon default prior simulations.

### Parameter estimation and Bayesian branch length estimates

**Figure 6:**
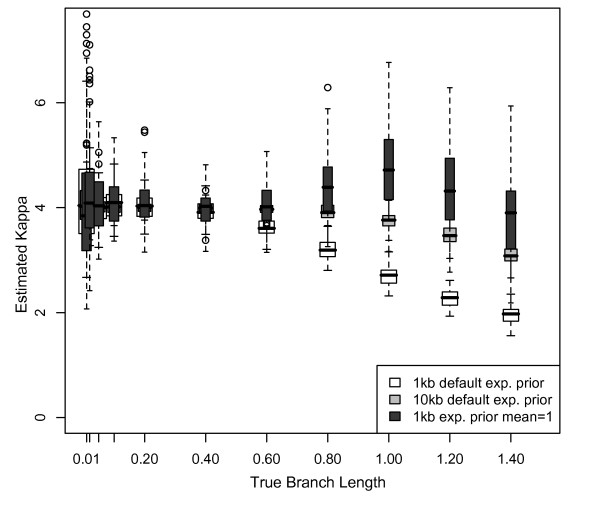
**Estimates of parameters in Bayesian analysis**. The estimated transition:transversion rate ratio (kappa) as a function of branch length. Kappa used for simulations was 4 (transition:transversion = 2, equal base frequencies, twice as many transitions as transversions). Kappa was estimated from the data in a Bayesian framework using MrBayes with the default exponential branch length prior (mean = 0.1) for 4-taxon, equal-branch-length, HKY 1 kb datasets (white boxes); with the default exponential branch length prior for 4-taxon, equal-branch-length, HKY 10 kb datasets (gray boxes); and with an exponential branch length prior of mean = 1 for 4-taxon, equal branch length, HKY 1 kb datasets (dark gray boxes).

Underestimation of branch length was correlated with underestimation of substitution model parameters, calculated as kappa (the transition/transversion ratio scaled by base frequencies) (Pearson's correlation coefficient = 0.343; P < 0.0001). A transition/transversion ratio of two, twice as many possible transitions as transversions, and equal base frequencies results in a kappa of four. Using the default exponential prior with mean equal to 0.1 with 1 kb datasets, kappa was estimated to be ~4 for branch lengths ≤ 0.4 substitutions/site (Figure 6 white boxes). For branch lengths ≥ 0.6, the estimate of kappa gradually decreased to 2 for branch lengths of 1.4 substitutions/site (Figure 6 white boxes). Parameter estimation was significantly better with larger datasets, with the estimated value of kappa for 10 kb datasets correct for branch lengths ≤ 0.8 and declining only to 3 for branch lengths of 1.4 substitutions/site when using the default prior (Figure 6 light gray boxes). In contrast, when using an exponential prior with mean equal to 1, kappa was estimated to be > 4 for branch lengths > 0.6, with the highest kappa at branch lengths of 1 substitution/site, decreasing to kappa = 4 for branch lengths of 1.4 substitutions/site (Figure 6 dark gray boxes). With a uniform prior, kappa was nearly identical to kappa for the default exponential prior.

### Bayesian branch length estimates under empirical conditions

**Figure 7:**
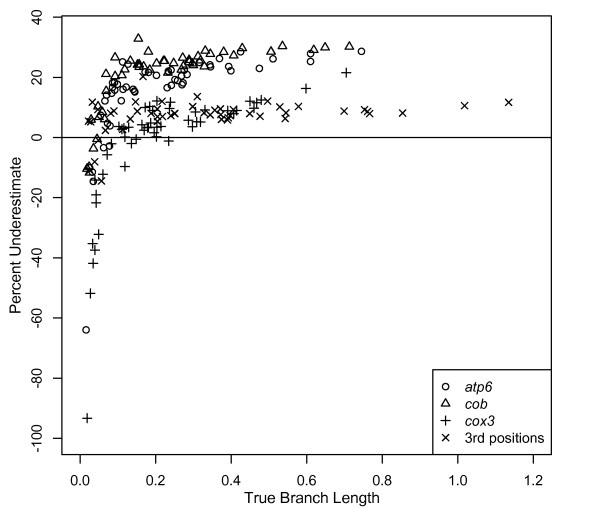
**Underestimate of Bayesian branch lengths using empirical parameters**. Percentage that branch lengths were underestimated for data simulated using empirical parameters for the mitochondrial genes *atp6*, *cob*, and *cox3*, as well as 3^rd ^codon positions for all 13 mitochondrial protein coding genes on the plethodontid salamander phylogeny of Mueller et al. (2004). Data were analyzed in a Bayesian framework using MrBayes to determine the effects of biologically realistic, unequal branch lengths on branch length estimation. For clarity, only the mean underestimate for each branch across simulations is shown.

The four sets of 100 datasets simulated with empirically estimated substitution models and branch lengths on the 27-taxon salamander phylogeny produced variably inaccurate, but generally poor, estimates of branch length. Of the 51 branches estimated, the lengths of just five branches for *atp6*, seven branches for *cob*, 32 branches for *cox3*, and 38 branches for 3^rd ^codon positions were estimated within 10% of the true branch length (Figure 7). Results were similar when branch lengths were randomized on the tree, suggesting no interaction between the length of branches and their position on the tree. This contrasts with results for 4- and 8-taxon HKY simulations, which showed a clear effect of branch depth. Longer branches (≥ 0.3) were underestimated at rates of 20-30% for *atp6 *and *cob*, and at rates of 5-20% for *cox3 *and 3^rd ^positions (Figure 7). Shorter branches (0.1-0.3) were underestimated at rates nearly the same as those for long branches --10-25% for *atp6 *and *cob *and 0-10% for *cox3 *and 3^rd ^codon positions (Figure 7). This result contrasts with results for 4- and 8-taxon HKY simulations, for which such branch lengths were estimated nearly correctly.

Compared to equal-branch-length 4- and 8-taxon HKY simulations, the lengths of long branches were significantly less underestimated and the lengths of shorter branches were significantly more underestimated. Both the lower-than-expected underestimation of long branches and the higher-than-expected underestimation of short branches are consistent with previous branch-length-heterogeneity results in this study. In the simple simulations with one long branch, the presence of shorter branches appeared to produce more accurate estimation of the longer branch. Similarly, in the simple simulations with one short branch, the presence of longer branches appeared to produce less accurate estimation of the shorter branch.

Base frequencies were estimated accurately for all of these analyses. The gamma shape parameter and the proportion of invariant sites were both significantly overestimated for all analyses; however, this pattern is likely the result of some sites that were assigned a very low substitution rate in simulations not experiencing substitutions. Such sites would have been counted in the proportion of invariant sites, thus increasing this estimate. Similarly, such sites would not have been counted when estimating the shape of the gamma distribution. Consequently, the shape parameter would be estimated as larger than the parameter used in simulations. These two changes likely compensate for one other, yielding a reasonable approximation of among-site rate heterogeneity, and have minimal impact on branch length estimates. The six r-matrix parameters were up to 26% over- or underestimated for the three genes; however, error for four of five estimated parameters for the 3^rd ^codon positions ranged from 9-99%. The final r-matrix parameter was simulated as zero, but estimated as 0.127.

### Branch length estimation using maximum likelihood

#### Branch length effects

**Figure 8:**
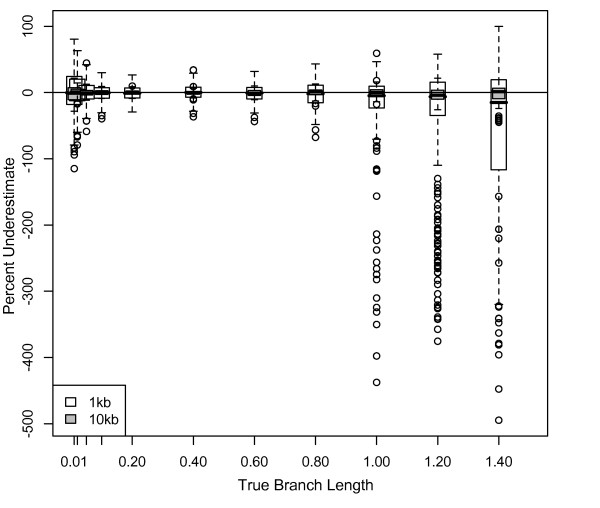
**Underestimate of ML branch lengths for 4-taxon trees**. Percentage that branch lengths were underestimated for 1 and 10 kb datasets simulated on 4-taxon trees with equal branch lengths using the HKY model with a transition/transversion ratio of 2 and equal base frequencies, and analyzed using maximum likelihood with parameters estimated from the data. This analysis is equivalent to that of Figure 2, but conducted using an ML framework; refer to the Figure 2 inset for the simulation topology.

For 4-taxon datasets, branch lengths ≤ 0.6 substitutions/site were estimated accurately for 1 kb datasets. However, for branch lengths ≥ 0.8 substitutions/site, lengths were increasingly overestimated (Figure 8 white boxes). For branch lengths of 1.4 substitutions/site, the median overestimate was 15%. This result contrasts with Bayesian results, for which the length of longer branches was significantly underestimated. As in the Bayesian framework, branch lengths were estimated more accurately for longer datasets (10 kb), with a median underestimate for branch lengths of 1.4 substitutions/site of only 1% (Figure 8 gray boxes).

#### Branch depth effects

**Figure 9:**
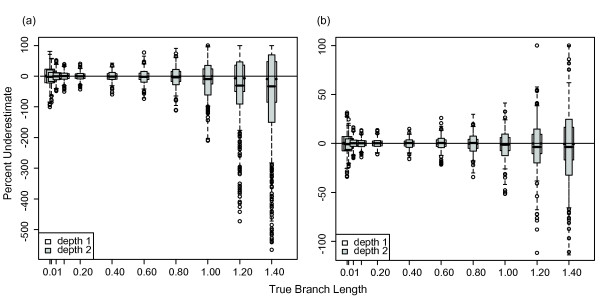
**Underestimate of ML branch lengths for 8-taxon trees**. Percentage that branch lengths were underestimated for datasets simulated on 8-taxon trees with equal branch lengths using the HKY model with a transition/transversion ratio of 2 and equal base frequencies, and analyzed using maximum likelihood with parameters estimated from the data. Depth 1 and depth 2 branches were graphed separately (white and gray boxes respectively. This analysis is equivalent to that of Figure 3, but conducted using an ML framework; refer to the Figure 3 inset for the simulation topology. (a) 1 kb datasets; (b) 10 kb datasets. Outliers (not shown) for depth 2 branches of 1.2 substitutions/site for 1 kb datasets were up to 30,000% overestimated (negatively underestimated) and were up to 50,000% overestimated for branch lengths of 1.4 substitutions/site.

Depth 2 branch lengths for 8-taxon 1 kb datasets (Figure 9a gray boxes) were more overestimated than depth 1 branch lengths for either 4- or 8-taxon datasets; the median overestimate for depth 2 branches of 1.2 and 1.4 substitutions/site was ~30%. Estimates of depth 1 branch lengths for 8-taxon 1 kb datasets (Figure 9a white boxes) were slightly better than for 4-taxon 1 kb datasets (Figure 8 white boxes); this result contrasts with Bayesian results, in which depth 1 branch length estimation error was consistent, regardless of the number of taxa. ML results were again significantly improved by the addition of data; for 10 kb datasets, depth 1 branches were estimated accurately (Figure 9b white boxes) and depth 2 branches were overestimated by < 4% (Figure 9b gray boxes).

#### Branch length heterogeneity effects

**Figure 10:**
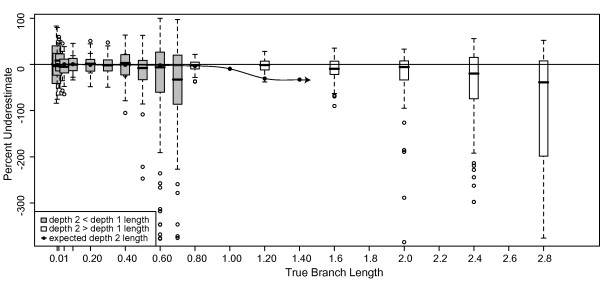
**Underestimate of ML branch lengths on unequal branch length trees**. Effects of unequal branch lengths on branch length estimation in a maximum likelihood framework. Results are plotted as for Figure 4. Gray boxes are the percent underestimation of depth 2 branch lengths for 4-taxon trees with the depth 2 branch length = half the depth 1 branch length. White boxes are the percent underestimation of depth 2 branch lengths for 4-taxon trees with the depth 2 branch length = double the depth 1 branch length (outliers of up to -30000% for branches of 1.4 substitutions/site are not shown for clarity). Depth 2 branch lengths were expected to be underestimated at the same rate as depth 2 branch lengths of 8-taxon equal-branch-length datasets (mean underestimation shown as filled circles). Half-length depth 2 branches (gray boxes) were generally overestimated (negatively underestimated) at a higher rate than expected (filled circles). Double-length depth 2 branches (white boxes) were overestimated at a lower rate than expected (filled circles and extrapolating from the trend of underestimation (spline interpolation line)).

Doubling or halving the length of the depth 2 branch of a 4-taxon tree (Figure 4 insets) had no effect on depth 1 branch length estimation. However, for longer branches (≥0.7 substitutions/site), depth 2 branch length estimates were affected by the degree of misestimation of the "majority" branch length (1.4 substitutions/site); in these cases, when the depth 2 branch length was halved, its length was more overestimated than expected based on the 8-taxon depth 2 results (Figure 10 gray boxes and filled circles). Conversely, when the length of the depth 2 branch was doubled it was less overestimated than expected based on the 8-taxon depth 2 results for longer branches (≥1.2 substitutions/site) (Figure 10 white boxes and filled circles). These results suggest that the rate of overestimation for the majority branch lengths exerts a "pull" of the rate of overestimation of the unique branch length, such that the unique branch length is overestimated at a rate more similar to the rate of overestimation for the other branches in the tree. These results mirror results for heterogeneous branch lengths estimated in a Bayesian framework.

### Effects of parameter estimation on maximum likelihood branch length estimates

**Figure 11:**
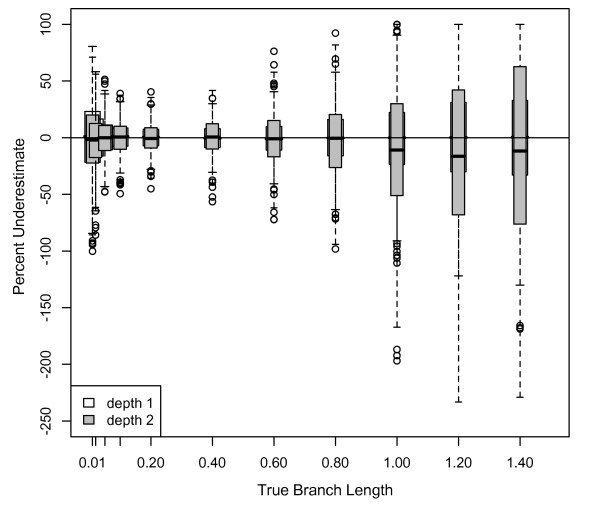
**Underestimate of ML branch lengths with fixed parameters**. Percentage that branch lengths were underestimated for 1 kb datasets simulated on 8-taxon trees with equal branch lengths using the HKY model with a transition/transversion ratio of 2 and equal base frequencies, and analyzed using maximum likelihood with fixed model parameters, Depth 1 and depth 2 branches are shown separately (white and gray boxes respectively). Open circles are outliers.

When parameters were fixed to those matching the substitution model used for simulations, the lengths of depth 1 branches for 4 and 8-taxon trees were estimated correctly for all dataset sizes (4-taxon results not show; Figure 11). However, even when parameter values were fixed to the simulation substitution model, the lengths of depth 2 branches were overestimated by 10-16% for the three longest branch lengths for 1 kb 8-taxon datasets (Figure 11). Such error was half of that obtained when model parameters were estimated. Again, results were significantly improved with the addition of data; both depth 1 and depth 2 branch lengths were estimated correctly in all cases for 10 kb datasets (results not shown). Thus, model parameter misestimation contributes to ML branch length misestimation for some combinations of taxon sampling, branch length, branch depth, and dataset size.

**Figure 12:**
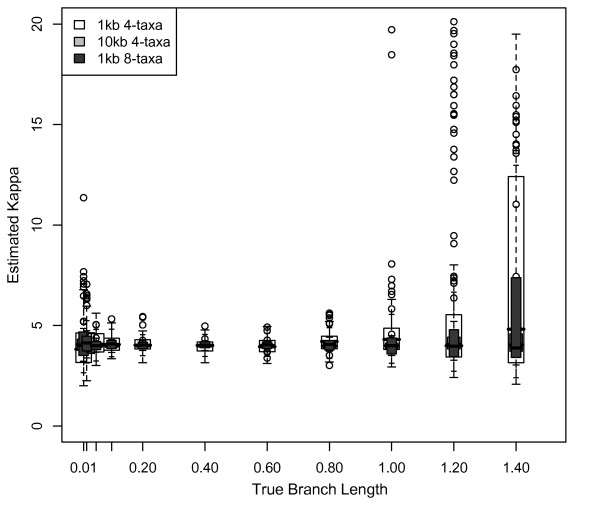
**Estimates of parameters in ML analysis**. The estimated transition:transversion rate ratio (kappa) plotted against branch length. Kappa was estimated from the data in an ML framework using PAUP* for 4-taxon equal-branch-length HKY datasets of 1 and 10 kb, and for 8-taxon datasets of 1 kb. Kappa used for simulations was 4 (transition:transversion = 2, equal base frequencies, twice as many transitions as transversions). This analysis is equivalent to that of Figure 6, but conducted using an ML framework.

When model parameters were not fixed, the median estimate of kappa was correct for 4-taxon datasets for branch lengths ≤ 1.2 substitutions/site, but overestimated (kappa = 4.8) for branch lengths of 1.4, with a wide range of estimates across 1 kb simulated datasets (Figure 12 white boxes). Parameter estimates were significantly improved by the addition of data, with correct median estimates of kappa for 10 kb datasets for all branch lengths (Figure 12 light gray boxes). The addition of taxa also resulted in correct median estimates for kappa for all branch lengths, with a smaller range of estimates of kappa across simulations compared to 4-taxon 1 kb datasets (Figure 12 dark gray boxes). The overestimation of kappa was correlated with overestimation of the length of depth 1 branches in ML analyses (Pearson's correlation coefficient = 0.826; P < 0.0001). In summary, both fixed- and unfixed-parameter results suggest that error in model parameter estimation contributes to maximum likelihood branch length misestimation in some simple simulations.

### Maximum likelihood branch length estimation under empirical conditions

**Figure 13:**
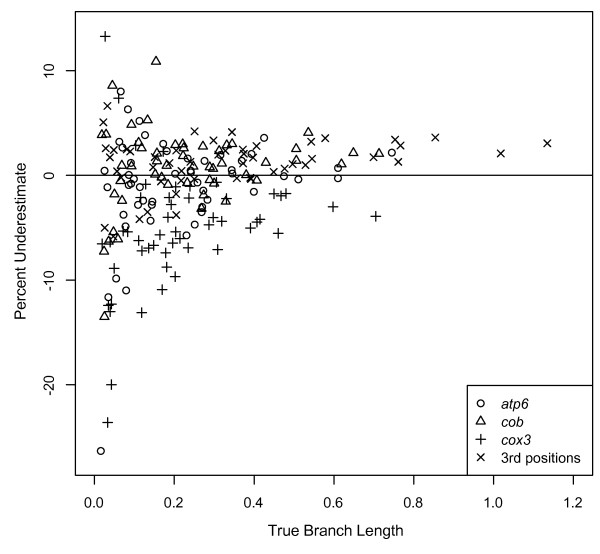
**Underestimate of ML branch lengths using empirical parameters**. Percentage that branch lengths were underestimated for data simulated using empirical parameters for the mitochondrial genes *atp6*, *cob*, and *cox3*, as well as the 3^rd ^codon positions for the 13 mitochondrial protein coding genes on the plethodontid salamander phylogeny of Mueller et al. (2004). Data were analyzed in an ML framework using PAUP* to determine the effects of biologically realistic, unequal branch lengths on branch length estimation. For clarity, only the mean underestimate for each branch across simulations is shown. This analysis is equivalent to that of Figure 7, but conducted using an ML framework.

Forty-three of 51 (84%) branch length estimates for data simulated on the 27-taxon salamander phylogeny for *atp6 *and *cob *were within 5% of the true branch length (Figure 13). When branch lengths were randomized on the tree, 43 and 41 estimates for *atp6 *and *cob*, respectively, were within 5% of the true branch length. All but two branches that were misestimated by > 5% for these genes were shorter than 0.2 substitutions/site (Figure 13). For *cox3*, length estimates for 43 of 51 branches were within 10% of the true value (Figure 13). When branch lengths were randomized on the tree, the lengths of 46 branches were within 10% of the true value. All branches that were misestimated by > 10% for this gene were shorter than 0.2 substitution/site (Figure 13). For 3^rd ^codon positions, length estimates for 47 of 51 branches were within 5% of the true value (Figure 13); results were identical when branch lengths were randomized on the tree. All branches that were misestimated by > 5% were shorter than 0.1 substitution/site (Figure 13). Overall, ML branch length estimates, even for long (> 0.3 substitutions/site), deep branches, were relatively accurate for all three genes. Even *cox3*, which has only 472 variable bases, performed relatively well on all but the shortest branches; however, the length of this gene may account for its higher error rate compared to the other partitions.

As with Bayesian analysis, base frequencies were estimated accurately for all of the partitions. The six r-matrix parameters were 1-3% misestimated for *atp6*, 7.5-17% for *cob*, 13.6-30% for *cox3*, and 1-4% for the four non-zero 3^rd ^codon position parameters (the remaining parameter was estimated as 0.01223 rather than 0). As in the Bayesian analysis, the gamma shape parameter and the proportion of invariant sites were both significantly overestimated for all analyses. Thus, as with simple simulations, branch lengths appeared to be estimated most accurately when parameters were estimated accurately. Surprisingly, when parameter values were specified to eliminate potential effects of parameter misestimation, results were similar to or worse than when parameters were estimated. Forty-one, 31, and 48 branch length estimates for *atp6, cob*, and 3^rd ^codon positions, respectively, were within 5% of the true branch length; 41 length estimates for *cox3 *were within 10% of the true value.

### Effects of erroneous branch lengths on divergence dating

**Figure 14:**
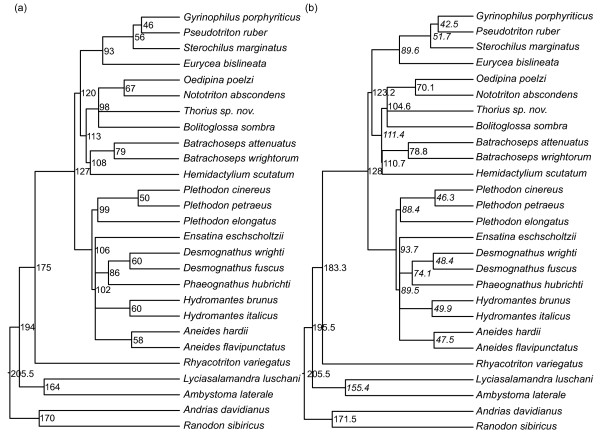
**Change in divergence date estimates for plethodontid salamanders following re-estimation of branch lengths using ML**. (a) Divergence dates for plethodontid salamanders estimated by Mueller (2006) using penalized likelihood, with branch lengths estimated using a Bayesian framework. (b) Divergence dates estimated in this study using penalized likelihood, with branch lengths estimated using ML. Italicized dates were estimated as younger than in the original analysis. Non-italicized dates were estimated as the same age or older than in the original analysis.

Because maximum likelihood estimates of branch lengths for the simulated "salamander" data were nearly identical to the true branch lengths, whereas Bayesian length estimates were significantly underestimated, we used a weighted average of ML branch length estimates to re-estimate plethodontid divergence dates in r8s. The divergence dates estimated by Mueller [37] using Bayesian branch lengths are shown in Figure 14a and the divergence dates estimated from this study are shown in Figure 14b. Recent nodes were estimated as younger than suggested by Mueller [37] by up to 20%, while older nodes were estimated as older by up to 7%.

## Discussion

### Comparison of Bayesian and ML results

Our 4- and 8-taxon simulation results suggest that, even for extremely simple trees, Bayesian branch lengths are misestimated; only a small range of branch lengths is estimated correctly. Above this range, branch lengths are progressively underestimated with increased branch length; below this range, branch lengths are progressively overestimated. Increasing underestimation with increasing branch length is consistent with the expected effects of site saturation - multiple hits are counted as single substitutions. Additionally, the prior distribution of branch lengths impacts branch length estimation. These results appear to conflict with previous work suggesting that Bayesian branch lengths are estimated correctly unless the model is under- or over-parameterized [39,40]. However, the range of branch lengths tested by Lemmon and Moriarty [39] was limited to the range for which we observed correct branch length estimation.

In contrast to our Bayesian results, the majority of ML branch length estimates are quite accurate for simple datasets, although some longer depth 2 branches are overestimated. ML misestimation of branch lengths produces different errors than those produced in a Bayesian framework; in ML, long branches are overestimated, whereas in a Bayesian framework, long branches are underestimated and short branches are overestimated. ML results are inconsistent with the expectation that longer branch lengths would be underestimated due to multiple hits counted as single substitutions. In both Bayesian and ML analyses, branch depth had a significant impact on estimation accuracy; deeper branches, as expected, were more misestimated than tip branches.

### Effects of model parameter misestimation

Bayesian branch length underestimation is explained, in part, by failure to account for multiple substitutions at some sites. Substitution models can suggest the presence of some of these substitutions; however, if the model itself is misestimated, then many substitutions will go undetected. In Bayesian analyses, kappa was increasingly underestimated as branch lengths increased, likely due to multiple, unobserved substitutions at some sites. For longer branch lengths, the greater frequency of transitions than transversions increases the likelihood that a site will have experienced two transitions, inferred as one, while a site with a transversion has a single substitution. Thus, as branch lengths increase, the transition/transversion ratio decreases, and branch lengths are underestimated. With larger datasets, parameter estimates and branch length estimates improved, as expected [41]. However, because it is not possible to specify the true parameters in MrBayes, an explicit quantification of parameter misestimation was not performed.

While branch lengths are generally estimated correctly in ML, long branch lengths are overestimated in simple simulated datasets, also likely due to model parameter misestimation. Kappa was increasingly overestimated as branch lengths increased, leading to overestimation of the number of transitions and total substitutions. The reasons for overestimation of kappa are unclear. Model parameters are estimated correctly for a broader range of branch lengths in ML than in Bayesian analyses; this may explain, at least in part, ML's superior performance at most branch lengths/depths. However, even when parameters are fixed in ML analyses of simple simulated datasets, the lengths of long, deep branches are significantly overestimated, suggesting that error remains in ML branch length estimation for some combinations of branch length, branch depth, and dataset size.

### Effects of priors in Bayesian analyses

In Bayesian analyses, the branch length prior also impacted branch length estimates. The impacts of the default exponential prior with mean of 0.1 matched expectations: branch lengths longer than the mean of the prior distribution were underestimated, and branch lengths shorter than the prior mean were overestimated. Thus, we predicted similar impacts for an exponential prior with mean of 1: overestimated branch lengths < 1 substitutions/site, underestimated branch lengths > 1 substitutions/site, and correct estimation of branch lengths ≈ 1 substitution/site. The shortest branch lengths were overestimated, as predicted. As branch lengths increased towards the prior mean, they were initially less overestimated, as expected. However, as branch lengths approached the prior mean, branch lengths remained overestimated, in contrast to expectation. To further evaluate this unpredicted overestimation, we repeated this analysis using a branch length prior with a mean of 1.4. As with the mean of 1, all branch lengths were overestimated, with short branch lengths estimated nearly correctly and longer branch lengths overestimated by 1-20% (results not shown). It is unclear in these cases why the effects of the branch length prior are unpredictable and generally result in overestimation of branch lengths.

When a uniform prior was used in analyses, branch lengths were generally estimated correctly within the bounds of the distribution (zero to one), although at the edges of the distribution, short branch lengths were overestimated and long branch lengths were underestimated. Thus, the prior contributed little to the posterior distribution. As branch lengths increased (above the prior distribution), underestimation increased, consistent with expectation and the results of the low-mean exponential prior. Longer branch lengths were more underestimated under a uniform prior than under an exponential prior, consistent with their lower probability under a uniform than exponential prior. This result suggests that a uniform prior can affect the posterior distribution if the bounds of the prior do not encompass the range of true branch lengths. When we repeated our analysis of 4-taxon datasets for branch lengths of 1.4 substitutions/site using a uniform prior with bounds of 0 to 1.5, branch lengths were less underestimated (median of 15% vs. 33%) than with a uniform prior with an upper bound of 1, as expected (results not shown).

### Effects of dataset complexity

At first glance, the results from our 4- and 8-taxon simulations on ultrametric trees with equal branch lengths suggest predictable patterns of misestimation for branches at multiple depths for both Bayesian and ML analyses. However, such patterns disappear with even a marginal increase in tree complexity; the presence of one branch of different length substantially impacts misestimation. Taken as a whole, our simple simulation results imply that (1) error exists in branch length estimation, both dependent on [30] and independent of model parameter misestimation; (2) error is generally less severe in an ML framework; and (3) although systematic effects of branch depth, branch length, and dataset size exist when analyzing simple simulated datasets, such error is unpredictable when combinations of different branch lengths exist, as is the case for empirical data. Results from our simulations in which branch lengths, branch length heterogeneity, dataset size, model parameters, and taxon sampling reflect empirical data from plethodontid salamanders are consistent with this; branch length misestimation in more complex datasets does not precisely mirror misestimation in simple simulations. However, the results for both simple and complex simulations are generally consistent for both Bayesian and ML analyses.

Based on Bayesian analyses of simple, heterogeneous-branch-length simulations (Figure 4), we expected that (1) high rates of underestimation for long branches and (2) low rates of underestimation for short branches would exert a combined "pull" on the overall rate of underestimation. This process would produce long branches that were less underestimated than expected from simple simulations, and short branches that were more underestimated. Results from complex "salamander" simulations were largely consistent with these predictions with the exception of very short branches, which were overestimated even more than in simple simulations.

Unlike our simple simulation results, we did not observe an effect of branch depth on branch length estimation in our "salamander" simulations; when branch lengths were randomized on the tree, longer branches were not more underestimated with increased depth. However, we note that this branch length randomization approach is not a thorough evaluation of this problem in more complex datasets because branch depth, and the associated opportunity for signal erosion, also depends on the length of the shallower branches. In sum, Bayesian results from more realistic simulations are generally consistent with simple simulations, but the complexity of the datasets produces specific effects not predicted from simple datasets.

In contrast, more complex datasets analyzed using ML resulted in an improvement in branch length estimation over simple simulated data. Even estimated branch lengths > 1 substitution/site (drastically overestimated in simple simulations) were quite accurate. Although branch lengths were significantly overestimated for depth 2 branches in simple simulations, such misestimation was nearly absent in more complex datasets. Long branches, estimated less accurately in simple simulations, were estimated accurately in complex simulations. However, short branches, estimated accurately in simple simulations, were estimated less accurately in complex simulations. Thus, as with Bayesian results, (1) the length of long branches was estimated more accurately than expected, (2) the length of short branches was estimated less accurately than expected, (3) there was no apparent effect of randomizing branch lengths on the tree, and (4) although results were generally consistent with simple simulations, dataset complexity led to specific effects not predicted from simple datasets.

The 4- and 8-taxon analyses in this study all rely on simple substitution models that (1) remain constant across sites and lineages, and (2) specify only a few parameters that can be estimated reasonably well from the data across at least some combinations of branch length and dataset size (Figures 6 and 12). In the simplest cases, model parameter (kappa) misestimation is strongly correlated with branch length misestimation. We expected a similar pattern for our complex simulations; however, model misestimation did not have nearly as much impact on branch length estimation for these datasets. When parameters were fixed to their true values in ML analyses, branch length estimation did not necessarily improve; in some cases, it actually became worse. In a Bayesian framework, the partition with the worst model estimates (3^rd ^codon positions) produced relatively accurate branch length estimates compared to other partitions with better-estimated models, although this may also reflect increased dataset length. Taken together, these results suggest that, although parameter and branch length estimation error were correlated in simple simulations, this correlation may have reflected a single underlying cause (such as insufficient data to estimate either parameter) rather than a causative relationship between model estimation and branch length estimation. Thus, the relationship between model misestimation and branch length misestimation in complex datasets warrants further research, particularly because the complex mutational processes producing real sequence diversity are never fully captured by nucleotide substitution models.

### Implications for empirical data collection and analysis

#### Dataset size

The majority of our analyses indicate that increased dataset size results in improved branch length estimates. However, the potential for increasing the size of empirical datasets to the point where the branch lengths may be estimated even within 10% in a Bayesian framework (e.g. >10 kb for depth 1 branch lengths >1.4 substitutions/site) is limited. Although next-generation sequencing enables the collection of vast amounts of data, mutations accumulate heterogeneously across the genome. Dataset partitions should be modeled individually to avoid error in phylogeny estimation reflecting the application of an average substitution model to multiple heterogeneous processes [42,43]. When the mitochondrial genome is partitioned by codon position, the longest partition is < 3.5 kb, and most nuclear introns are < 5 kb; our results suggest that such datasets are insufficient to obtain accurate Bayesian branch length estimates. However, for ML analyses, even the shortest empirically-based dataset we tested (< 500 variable bases) had 84% of branches estimated within 10% of the true length; for larger datasets (516-3638 bp), 84% were estimated within 5%.

#### Analytical framework

Our results suggest that error remains in branch length estimation, both dependent on and independent of substitution model parameter misestimation, given dataset sizes comparable to many empirical studies. Such error appears more pronounced in Bayesian than ML analyses. Branch length prior affects topology estimation [44,45]; therefore, our finding that branch length prior impacts branch length estimation is not surprising. This study is limited to the estimation of branch lengths on a known phylogeny; we do not suggest that ML is the most accurate method of phylogenetic inference overall. Numerous other studies have addressed methods for estimating phylogenies correctly [e.g. [45-47]]. For example, Mar et al. [45] suggested that phylogenetic inference in a Bayesian framework may be more robust than ML when there is significant variation among branch lengths. However, our results suggest that branch lengths are more accurately estimated using ML than Bayesian analysis.

### Implications for divergence dating

Previous work has shown that ML and Bayesian analyses can yield different divergence date estimates [e.g. [2,48]], but such comparisons have not suggested which result is likely to be more accurate. We found that both ML and Bayesian branch length estimates are subject to error, but that ML estimates are more accurate, given realistic datasets. Additionally, the most substantial error in ML analyses is associated with short branches, which have less effect on divergence dating than do long branches because the age of each node is based on its depth in the tree. With the exception of studies with extremely dense taxon sampling, node depth generally reflects the sum of fewer, longer branches rather than numerous, short branches.

In light of these results, we repeated a Bayesian analysis of plethodontid salamander divergence dates using ML. This reanalysis resulted in significant changes in divergence dates: shallow nodes were estimated as younger than previously suggested, while deeper nodes were estimated as slightly older. This pattern was expected because long branches that were underestimated in a Bayesian framework were corrected, resulting in an increase in the estimated number of substitutions/site/million years. In this case, the primary fossil calibration was fixed on a long branch; branches of similar length were corrected at the same rate such that the ages of older nodes were not significantly affected by this reanalysis. However, the length of short branches was similar in a Bayesian and ML framework; therefore, an increase in the estimated average substitution rate results in younger divergence dates for shallow nodes [28]. Our reanalysis demonstrates the magnitude of potential effects of Bayesian branch length misestimation on divergence date estimates. Other studies that also utilized Bayesian branch length estimates in a penalized likelihood analysis of divergence dates [49] may have incurred similar error and be appropriate targets for a similar reanalysis. However, we note that the substantial confidence intervals associated with many divergence date estimates likely accommodate much of the error from inaccurate branch length estimates. Additionally, we note that many other factors, including fossil data and analytical tools used, affect the accuracy of divergence date estimation [[12,50,51], e.g. [52,53]].

Finally, various alternative methods have been proposed to estimate divergence dates, which are affected by our results to varying degrees. Other rate smoothing procedures [e.g. [54]] will be similarly affected by branch length misestimation. Bayesian methods, such as BEAST [55] and multidivtime [20] may also be affected; BEAST and MrBayes share the underlying core MCMC algorithm, which is used to identify high likelihood trees [55]. However, in BEAST and multidivtime, substitution rates for each branch are estimated using a relaxed clock approach, which may limit the effects of overestimation of short branches; because substitution rates are drawn from a distribution, the probability of high rates on short branches is greatly reduced. BEAST also uses prior distributions on node dates and mutation rates, rather than on branch lengths.

## Conclusions

Divergence date estimation has long been one of the goals of phylogenetic systematics. Error in divergence date estimation due to error in branch length estimation can result in flawed conclusions about molecular evolution and historical environmental events leading to speciation. In this study, we found that accuracy of branch length estimation is affected by the length of the dataset, the length of the branch and of the other branches in the tree, the depth of the branch, and the statistical framework in which branch lengths are estimated. We suggest that branch lengths can be estimated most reliably in an ML framework when branches are <1 substitution/site and datasets are = 1 kb. Divergence date estimates using datasets, branch lengths, and/or analytical techniques that fall outside of these parameters should be interpreted with caution.

## Methods

### Baseline Bayesian branch length estimates

To determine the accuracy of Bayesian branch length estimates across a range of branch lengths, we conducted initial simulations using the HKY model of evolution [56] with a transition/transversion ratio of 2 and equal base frequencies. One hundred datasets of 1 kb each were simulated in SeqGen [57] on balanced four-taxon trees (Figure 1a) with equal branch lengths of 0.01, 0.02, 0.05, 0.1, 0.2, 0.4, 0.6, 0.8, 1.0, 1.2, and 1.4 substitutions/site. An unrooted phylogeny (including the tree topology and branch lengths) for each dataset was estimated in a Bayesian framework in MrBayes 3.2 [58] with two MCMCMC chains and an HKY substitution model with parameters estimated from the data. Each chain was run for one million generations with trees sampled every 100 generations. The first 3000 trees (30%) were discarded as burn-in and the remaining trees were summed to determine the consensus phylogeny and branch lengths (Figure 1b). The average standard deviation of split frequencies was checked for a subsample of runs for each analysis to ensure it was < 0.01; this is the suggested diagnostic for determining when the different runs will produce a good sample from the posterior probability distribution. Burnin was determined based on the suggested 25%, which is assumed when the convergence diagnostic is calculated. The burnin was rounded up to 30% to be more conservative in excluding low probability trees. The graph of negative log likelihoods was also examined for a subsample of analyses to ensure that this value had stabilized for both runs, suggesting that the selected burnin had removed low probability trees. For each simulation, the length of each branch was calculated in MrBayes as the mean of its length for each sampled tree (after the burnin) for which the correct bipartitions associated with that branch were found. All consensus trees successfully recovered the correct tree topology. To determine branch length estimation accuracy, the estimated length of all branches for each set of simulations was compared to the known branch length. Only depth 1 branches were included in this analysis to avoid the potentially complicating factor of node depth (Figure 1a,b). The percentage by which each depth 1 branch was underestimated was graphed as a function of true branch length using R [59]. We used a boxplot to show the full range of underestimates across all simulations. To ensure that 100 simulations was sufficient to produce consistent results, the results for sets of 100 datasets were compared to the results for a subsample of 50 datasets to ensure that the two were the same.

### Effects of dataset size on Bayesian branch length estimates

We repeated the previous analysis with 100 datasets of 10 kb to determine whether branch lengths are estimated more accurately with longer datasets. Longer datasets (1) provide more data with which to estimate of the number of substitutions per site, and (2) increase the accuracy of model parameter estimates and, thus, the accuracy of estimating multiple substitutions.

### Effects of number of taxa and branch depth on Bayesian branch length estimates

Previous work has suggested that deeper branches are more likely to be affected by model misspecification because of erosion of phylogenetic signal in the descendant lineages [e.g. [28]]. To determine whether branch length estimation is affected by (1) the depth of the branch in the tree, and (2) the number of taxa in the tree, we repeated the previous simulations of 100 1 kb datasets on balanced 8-taxon trees of equal branch lengths spanning 0.01 - 1.4 substitutions/site (Figure 1c). These simulations differ from the 4-taxon simulations of identical branch length in two ways: (1) there are twice as many taxa in the dataset, and (2) branches are positioned at three different depths in the tree (two of which have multiple samples). Incorrect topologies were estimated for 15 datasets of 1.0 substitutions/site, 44 datasets of 1.2 substitutions/site, and 62 datasets of 1.4 substitutions/site; these data were removed from further analysis. We compared branch length estimation accuracy for depth 1 and depth 2 branches separately. Additionally, estimation accuracy of depth 1 branches for 4- and 8-taxon trees was compared to determine effects of the addition of taxa. To ensure that the number of simulations was sufficient to produce consistent results, the results for all datasets were compared to the results for a subsample of 50 datasets.

### Effects of branch length heterogeneity on Bayesian branch length estimates

Our previous simulations were conducted using identical branch lengths for all branches in the tree, which is an unrealistic situation. To determine whether interactions among different branch lengths affect branch length estimation accuracy, we repeated the previous 100 4-taxon simulations with the depth 2 branch of the tree either half or double the length of the other branches (Figure 1d). We varied the length of this branch (as opposed to a terminal branch) to retain an ultrametric tree such that results from this analysis would be comparable to results from other analyses in this study, which were also performed on ultrametric trees. In the absence of interactions among branch lengths, this branch would be misestimated similar to depth 2 branches of the same length in 8-taxon simulations. Incorrect topologies were estimated for 1 dataset of 1.0 substitutions/site, 6 datasets of 1.2 substitutions/site, and 8 datasets of 1.4 substitutions/site with half-length middle branches; these data were removed from further analysis.

### Effects of branch length prior on Bayesian branch length estimates

Bayesian analysis incorporates some prior prediction of the distribution of branch lengths. In all previous analyses in this study, we used an exponential prior with mean equal to 0.1. This exponential prior for branch lengths is the default in MrBayes because a uniform prior has been suggested to result in overestimated posterior probabilities for clades [44]. To determine whether this prior affects branch length estimates, we repeated our previous analyses of the 100 4-taxon datasets simulated on equal branch length trees, specifying both an exponential prior with mean equal to 1 and a uniform prior (lower bound of 0, upper bound of 1). Incorrect topologies were estimated for 1 dataset of 1.4 substitutions/site analyzed with an exponential prior of mean 1, 14 datasets of 1.0 substitutions/site analyzed with a uniform prior, and 41 datasets of 1.2 substitutions/site analyzed with a uniform prior; these data were removed from further analysis.

### Effects of parameter estimation on Bayesian branch length estimates

Incorrect parameter values in substitution models are known to affect branch length estimation [28,31,60]. We examined parameter estimates for each of the 100 4- and 8-taxon simulated datasets to determine whether such parameters were estimated correctly. We then evaluated the extent to which parameter misestimation was correlated with branch length and dataset size. Finally, we used a Pearson's correlation coefficient to determine whether error in branch length estimation was correlated with error in parameter estimation. For simplicity, this correlation was limited to results for 4-taxon datasets of 1 kb and 10 kb using the default prior, and 1 kb with an exponential prior of mean equal to 1.

### Bayesian branch length estimates under empirical conditions

Using empirical trees as the basis for simulations provides an estimate of the combined effects of (1) complex model estimation using realistic datasets, (2) complex distribution of branch lengths, and (3) multiple tree depths on rates of branch length underestimation. Such empirically-based analyses address the extent to which our simple simulation results can be generalized to empirical data. To this end, we conducted simulations on the 27-taxon phylogeny for plethodontid salamanders determined by Mueller et al. [38]. We used the GTR+I+Γ parameters previously identified for each of three mitochondrial genes (*atp6, cob, cox3*) and the combined 3^rd ^codon positions for all 13 mitochondrial protein-coding genes using AIC implemented in Modeltest [37]. Parameters for the three genes and the combined 3^rd ^codon positions ("partitions" hereafter) are provided in Table 1. Branch lengths for each simulation tree (one for each partition) were estimated in MrBayes using the empirical data and a constrained topology [38]. Although the branch length estimates and model parameters on which we based these simulations are imperfect with respect to reality, they represent a step towards biological realism.

**Table 1:** Parameters used to simulate realistic datasets on the plethodontid salamander phylogeny

Gene	Base frequencies (pi(A), pi(C), pi(G), pi(T))	R Matrix (A⇔C, A⇔G, A⇔T, C⇔G, C⇔T, G⇔T)	Gamma shape	P invar	Length (bp)	Max branch length
*atp6*	0.397 0.230 0.061 0.312	0.396 6.362 0.323 0.276 4.258 1.0	0.4908	0.2436	683	0.75
*cob*	0.407 0.236 0.063 0.294	1.0 10.749 1.0 1.0 27.120 1.0	0.3611	0.2980	1141	0.71
*cox3*	0.403 0.228 0.072 0.297	0.620 10.185 0.632 0.694 20.706 1.0	0.4079	0.3985	784	0.70
3^rd ^positions	0.478 0.194 0.054 0.274	0.118 6.885 0.109 0.000 6.205 1.0	0.7208	0.0063	3661	1.13

One hundred datasets corresponding to each partition were simulated on the phylogeny with corresponding branch lengths. Because branch length and depth in the tree were correlated in this empirical dataset -- all the long branches were at the tips of the tree -- we also randomized the branch lengths for each partition on the tree to avoid confounding branch length and depth. We simulated 100 additional datasets on these "randomized" trees using the same parameters. Branch lengths were estimated for each simulated dataset in MrBayes, using the constrained topology, to allow comparisons between estimated and known branch lengths. We evaluated the accuracy of parameter estimates for each dataset to identify potential correlations between parameter misestimation and branch length misestimation.

### Branch length estimation using maximum likelihood

Previous work has suggested that divergence date estimates can vary depending on the statistical framework used (i.e. Bayesian or maximum likelihood) [61]. All previous analyses (with the exception of variation in the prior) were repeated using maximum likelihood in PAUP* [62]. The topologies for these analyses were fixed, the general models used for simulations were specified (HKY for 4- and 8-taxon simulations, and GTR+I+Γ for the empirically-based 27-taxon "salamander" simulations), and parameters were estimated from the data. Because maximum likelihood does not incorporate a prior distribution, any effects of the prior on branch length estimates seen in Bayesian analyses should not be observed using ML.

### Effects of parameter estimation on maximum likelihood branch length estimates

We analyzed potential effects of parameter misestimation using two approaches. First, the evolutionary model and model parameters were fixed to those used for simulations, and analyses of the 100 simulated 4- and 8-taxon datasets were repeated using ML. Similarly, the evolutionary model and model parameters were fixed to the model used for "salamander" simulations and the data simulated on the salamander tree was analyzed using ML. These analyses eliminated any potential problems associated with model misestimation, isolating the remaining effects of dataset, branch length and depth, and branch length heterogeneity across the tree on branch length estimation. Second, for the simulated 4- and 8-taxon datasets and the empirically-based "salamander" simulations, we allowed parameters to be estimated from the data and evaluated the extent to which (1) misestimation of parameters was correlated with branch length and dataset size, and (2) parameter misestimation was correlated with branch length misestimation. As in the Bayesian analysis, the latter effect was evaluated using a Pearson's correlation coefficient.

### Effects of erroneous branch length estimates on divergence dating

To determine the effects of erroneous branch length estimates on divergence date estimates, we reanalyzed the mitochondrial genomic data of Mueller [37] for plethodontid salamanders, which used Bayesian estimates of branch lengths for penalized likelihood estimates of divergence dates. Methods were duplicated, with data partitioned by codon position (3603 bp each), ribosomal genes (957 and 725 bp), and concatenated tRNAs (1282 bp). However, to avoid potential error in branch length estimates suggested by results from this study, we used the analytical method suggested to be more accurate -- ML. Parameters of the GTR+I+Γ substitution model were estimated for each partition, and maximum likelihood branch lengths for each partition were estimated on the topology of Mueller [37] in PAUP*. A weighted average of branch lengths estimated for each partition was used to re-estimate divergence dates via penalized likelihood [17] in r8s [63], with the identical fossil calibrations used by Mueller [37].

## Authors' contributions

Both authors contributed to the conception and design of this study, and to the writing of this paper. Both authors read and approved the final manuscript. RSS carried out the simulations and analyses.
